# Genomic Analysis of *Plasmodium vivax* in Southern Ethiopia Reveals Selective Pressures in Multiple Parasite Mechanisms

**DOI:** 10.1093/infdis/jiz016

**Published:** 2019-01-21

**Authors:** Sarah Auburn, Sisay Getachew, Richard D Pearson, Roberto Amato, Olivo Miotto, Hidayat Trimarsanto, Sha Joe Zhu, Angela Rumaseb, Jutta Marfurt, Rintis Noviyanti, Matthew J Grigg, Bridget Barber, Timothy William, Sonia Morgado Goncalves, Eleanor Drury, Kanlaya Sriprawat, Nicholas M Anstey, Francois Nosten, Beyene Petros, Abraham Aseffa, Gil McVean, Dominic P Kwiatkowski, Ric N Price

**Affiliations:** 1 Global and Tropical Health Division, Menzies School of Health Research and Charles Darwin University, Darwin, Northern Territory, Australia; 2 Centre for Tropical Medicine and Global Health, Nuffield Department of Clinical Medicine, University of Oxford, United Kingdom; 3 College of Natural Sciences, Addis Ababa University, Addis Ababa, Ethiopia; 4 Armauer Hansen Research Institute, Addis Ababa, Ethiopia; 5 Wellcome Sanger Institute, Hinxton, Cambridge; 6 Big Data Institute, Li Ka Shing Centre for Health Information and Discovery, Oxford, United Kingdom; 7 Mahidol–Oxford Tropical Medicine Research Unit, Mahidol University, Bangkok, Thailand; 8 Eijkman Institute for Molecular Biology, Jakarta, Indonesia; 9 Agency for Assessment and Application of Technology, Jakarta, Indonesia; 10 Infectious Diseases Society, Sabah-Menzies School of Health Research Clinical Research Unit, Sabah, Malaysia; 11 Clinical Research Centre, Queen Elizabeth Hospital, Sabah, Malaysia; 12 Jesselton Medical Centre, Sabah, Malaysia; 13 Shoklo Malaria Research Unit, Mahidol–Oxford Tropical Medicine Research Unit, Faculty of Tropical Medicine, Mahidol University, Mae Sot, Thailand

**Keywords:** malaria, *Plasmodium*, vivax, genomics, Ethiopia, Duffy

## Abstract

The Horn of Africa harbors the largest reservoir of *Plasmodium vivax* in the continent. Most of sub-Saharan Africa has remained relatively vivax-free due to a high prevalence of the human Duffy-negative trait, but the emergence of strains able to invade Duffy-negative reticulocytes poses a major public health threat. We undertook the first population genomic investigation of *P. vivax* from the region, comparing the genomes of 24 Ethiopian isolates against data from Southeast Asia to identify important local adaptions. The prevalence of the Duffy binding protein amplification in Ethiopia was 79%, potentially reflecting adaptation to Duffy negativity. There was also evidence of selection in a region upstream of the chloroquine resistance transporter, a putative chloroquine-resistance determinant. Strong signals of selection were observed in genes involved in immune evasion and regulation of gene expression, highlighting the need for a multifaceted intervention approach to combat *P. vivax* in the region.

## Teaser

Genome-wide signatures of selection were investigated in an Ethiopian *Plasmodium vivax* population. Antimalarial drug pressure appeared lower than in Asia, but there was a high prevalence (80%) of Duffy binding protein amplifications, potentially reflecting adaptation to Duffy-negative red blood cells.

**(See the Editorial commentary by Sibley, on pages 1716–8.)**

Reports of drug resistance and severe disease have refocused efforts to contain *Plasmodium vivax*, with almost 3 billion people living at risk of infection [1]. However, this species has proven to be highly resilient, with a high capacity for resurgence, and extensive genetic diversity providing a rich baseline for adaptation to local selective pressures [2–5].

Although the largest burden of *P. vivax* is in South and Southeast Asia, the Horn of Africa harbors 10%–20% of all cases, presenting an important reservoir of infection in Africa [1, 6]. Ethiopia suffers the largest number of clinical cases in the region [6], partly reflecting the large percentage of Duffy-positive individuals [7]. For several decades, the central dogma has been that the host Duffy antigen is essential for *P. vivax* to invade host red blood cells (RBCs) and thus establish a blood-stage infection [8]. However, recent reports have demonstrated the emergence of strains able to invade Duffy-negative RBCs, raising concern over the potential spread of vivax into the large regions of sub-Saharan Africa, which were previously assumed to be protected by high proportions of Duffy negativity [9].

Microsatellite-based studies of *P. vivax* in Ethiopia have revealed extensive parasite diversity [10–12], underpinning a high capacity for local adaptation to host and environmental challenges such as Duffy negativity or antimalarial drug pressure. However, while several population genomic studies have been undertaken in vivax populations from Asia, the Pacific, and South America [3, 5, 13–17], none has explored the genomes of *P. vivax* from the horn of Africa.

We used whole genome sequencing data to investigate adaptive molecular changes in an Ethiopian *P. vivax* population. Comparisons were undertaken against existing genomic data from Southeast Asian *P. vivax* populations to identify Ethiopian-specific adaptive changes.

## METHODS

### Ethical Considerations

Ethical approval for patient sampling in Ethiopia was granted by the ethics boards of the Addis Ababa University College of Natural Sciences (RERC/002/05/2013), the Armauer Hansen Research Institute (AHRI-ALERT P011/10), the National Research Ethics Committee of Ethiopia (3.10/580/06), and the Human Research Ethics Committee of Northern Territory Department of Health and Families, Australia (HREC-13–1942). Written informed consent was obtained from all participants or a legal guardian where participants were ≤18 years of age.

### Study Site

The Ethiopian samples were sourced within the framework of previously described surveys recruiting symptomatic patients with *P. vivax*–positive blood smears [10]. Samples were collected between May and November 2013 from 4 sites in southern Ethiopia; Arba Minch, Misrak Badowacho, Halaba, and Hawasa (Supplementary Figure 1). Details on the local malaria epidemiology are published elsewhere [10]. In brief, *Plasmodium falciparum* and *P. vivax* are the dominant malaria-causing species, with vivax proportions ranging from 28% to 55%. The annual *P. vivax* parasite incidence (API) in 2012 ranged from 20–82 cases per 1000 population across the sites. The national policy for treating uncomplicated *P. vivax* infection is chloroquine plus primaquine, whereas artemether-lumefantrine is used to treat mixed-species infections of *P. falciparum* and *P. vivax*.

For comparative analysis, previously published genomic data were derived from Thailand, Indonesia, and Malaysia [5, 13]. These samples were sourced from symptomatic patients with *P. vivax* infection attending outpatient clinics in Tak Province, Thailand (2006–2013); Mimika district, Papua Indonesia (2011–2014); and Sabah, Malaysia (2010–2015). Estimates of the *P. vivax* incidence (API per 1000 population) at these times were 15.5 in Tak Province, 69.2–148.7 in Mimika district, and 0.02–0.32 in Sabah (data from Malaria Atlas Project and Sabah Department of Health). The first-line policy for treating *P. vivax* infection at the time of the enrollments was chloroquine plus primaquine in Thailand and Malaysia, and dihydroartemisinin-piperaquine plus primaquine in Indonesia.

### Sample Processing

Patient sampling involved collection of 2–5 mL venous blood, with leukocyte depleted by cellulose filtration [18]. DNA extraction was performed using commercial kits (Qiagen), and *Plasmodium* species was confirmed by polymerase chain reaction [19, 20].

### Whole Genome Sequencing

Leukocyte-depleted samples with ≥50 ng total DNA comprising <90% human DNA were subject to whole genome sequencing, read alignment, and variant calling within the framework of a *P. vivax* community study in the Malaria Genomic Epidemiology Network (MalariaGEN) [21]. Details on the library preparation, sequencing, and variant calling procedures are provided elsewhere [5]. In brief, sequencing was undertaken on the Illumina Hi-Seq platform using the manufacturer’s protocol for paired-end 150-bp reads. Reads were aligned against the *P. vivax* P01 reference [22] using bwa-mem version 0.7.15 [23], and single-nucleotide polymorphism (SNP) discovery and genotype calling were undertaken using previously defined methods [5], with data derived from the MalariaGEN *P. vivax* Genome Variation Project release 3.0. A set of 827 902 high-quality bi-allelic SNPs with a variant quality score log-odds >0 and <5% missing calls in high-quality samples (samples with ≥95% calls) were derived from an initial set of 4 084 419 discovered variants. Positions with <5 reads were defined as missing calls. For haplotype-based analyses, either the major or reference allele (at positions with equal allele depths) was used at heterozygote positions. Large copy number variations (CNVs) >3 kbp were detected using a hidden Markov model in pysamstats (http://github.com/alimanfoo/pysamstats) as described previously [24]. As the accuracy of the hidden Markov model in predicting the breakpoint sequence is imperfect, closer inspection of CNV boundaries was undertaken using Artemis software. The genomic data on the previously published and the new Ethiopian isolates are available in the European Nucleotide Archive (see [5, 13] and Supplementary Table 1, respectively).

### Data Analysis

Within-host infection complexity was assessed using the within-sample *F* statistic (*F*_WS_) [25, 26]. A threshold of *F*_WS_ > 0.95 was used as a proxy to monoclonal infection. Ethiopian isolates with *F*_WS_ < 0.95 were deconvoluted (phased) using DEploid identity by descent (IBD) [27] to determine the within-sample relatedness. DEploid-IBD was run with the default settings using a panel of 4 distinct Ethiopian isolates with *F*_WS_ > 0.95 (QS0002-C, QS0018-C, QS0028-C, and QS0042-C). Downstream haplotype-based analyses were undertaken on the samples with *F*_WS_ > 0.95, and additionally explored with the deconvoluted infections. Infection complexity was also assessed using the proportion of runs of homozygosity (RoH) in the isolates from all populations using previously described methods [5].

The number of nucleotide differences between pairs of DNA sequences was calculated at the SNP positions using VCFtools version 0.1.13 [28] and divided by the number of nucleotides in the core genome (21 310 118 bp) to derive an approximation of the genome-wide average nucleotide diversity (*pi*) for each population. Neighbor-joining (NJ) and principal coordinates analyses (PCoA) were conducted using a pairwise distance matrix calculated using R, and NJ plots were created using iTOL software [29].

Tajima’s *D* was calculated using the scikit-allel package (https://github.com/cggh/scikit-allel). Only genes with ≥10 SNPs across all populations were assessed. Pairwise measures of the genetic differentiation (fixation index [*F*_ST_]) at individual SNPs were calculated using the Weir and Cockerham formula [30]. The PlasmoDB Gene Ontology (GO) analysis tool was used to investigate enrichment of biological processes and molecular functions among the genes within the 1st and 99th percentile of the Tajima’s *D* distributions in Ethiopia and highly differentiated variants between Ethiopia and Asia (http://plasmodb.org).

The *Rsb* measure of cross-population extended haplotype homozygosity and the integrated haplotype score (iHS) were measured using the R-based *rehh* package [31]. For iHS analysis, ancestral alleles were derived by mapping *Plasmodium cynomolgi* reads [32] against the *P. vivax* P01 reference. Only positions where *P. cynomolgi* calls were homozygote and matched either the reference or alternative *P. vivax* allele were included in analysis.

Owing to extensive population structure in Malaysia, iHS, *Rsb*, and Tajima *D* were not investigated in this population, and *F*_ST_ results between Ethiopia and Malaysia were explored secondary to results against Thailand and Indonesia.

## RESULTS

### Genomic Data Summary

A total of 293 samples exhibited <5% missing genotype calls at 827 902 high-quality SNPs with genotype failure rates <5%. These samples include 24 new isolates from Ethiopia (detailed in Supplementary Data 1), and previously described samples from Thailand (n = 104), Indonesia (n = 111), and Malaysia (n = 54) [5, 13].

### Comparable Superinfection and Cotransmission Rates in Ethiopia, Indonesia, and Thailand

**Figure 1.**
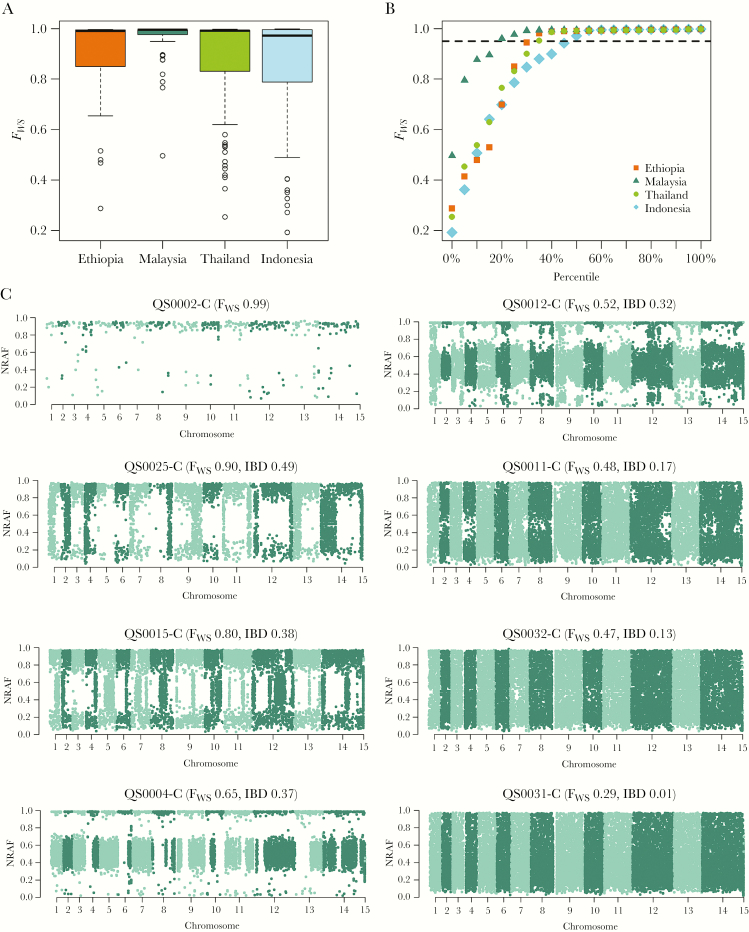
Within-sample infection complexity in Ethiopia relative to the Southeast Asian populations and within polyclonal Ethiopian infections. The boxplots (*A*) and scatterplots (*B*) illustrate the distribution of within-sample *F* statistic (*F*_WS_) scores in Ethiopia relative to Thailand, Indonesia, and Malaysia. Data are presented on all 293 high-quality samples. *B*, Dashed line illustrates *F*_WS_ = 0.95, above which infections are essentially monoclonal. Ethiopia exhibits an intermediate proportion of monoclonal infections relative to Malaysia and Indonesia, most comparable to Thailand. *C*, Manhattan plots of the nonreference allele frequency (NRAF) in 7 Ethiopian infections identified as polyclonal based on *F*_WS_ < 0.95, and 1 monoclonal infection as a baseline reference (QS0002-C). Trends in the pairwise identity by descent (IBD) between clones in a given infection (as determined by DEploid-IBD) correlated positively with the *F*_WS_ scores and approximated runs of homozygosity (RoH, not presented). DEploid-IBD found evidence of 2 major clones in QS0025-C, QS0015-C, and QS0004-C, and 3 clones in QS0012-C, QS0011-C, QS0032-C, and QS0031-C. The clones within QS0025-C demonstrated the highest pairwise IBD (0.49), indicative of siblings sharing approximately 50% of their genomes, as illustrated by the long stretches of homology (regions with NRAF approximating 0 or 1) on each chromosome. In striking contrast, the clones within QS0031-C exhibited the lowest pairwise IBD (<0.01%), with no evidence of recent common ancestry, rather reflecting a probable superinfection.

The proportion of monoclonal infections in Ethiopia (71% [17/24]) was similar to Indonesia (52% [58/111]; *P* = .151), Thailand (65% [68/104]; *P* = .787), and Malaysia (81% [44/54]; *P* = .451), although the latter displayed a skew toward higher *F*_WS_ scores (Figure 1A and 1B). DEploid-IBD analysis of the 7 polyclonal Ethiopian samples revealed that 3 and 4 infections had 2 and 3 major clones (>10%), respectively. Four (57%) of the polyclonal infections displayed IBD >25% in pairwise comparisons of the clones, suggesting relatedness at the half-sibling level or greater, and likely reflecting cotransmission rather than superinfection (multiple inoculations either acquired within a few weeks of one another or over several months with hypnozoites; Supplementary Data 2). The remaining 3 infections exhibited IBD ranging from 0 to 17%, more consistent with superinfections (Figure 1C). There was no significant difference in the median RoH proportions in the polyclonal infections in Ethiopia (0.03 [interquartile range {IQR}, 0.01–0.51]) relative to Thailand (0.08 [IQR, 0.005–0.49]; *P* = .779) or Indonesia (0.28 [IQR, 0.03–0.54]; *P* = .519), suggesting similar rates of superinfection vs cotransmission in these populations (Supplementary Figure 2; Supplementary Data 2). However, median RoH was significantly higher in the preelimination setting of Malaysia (0.73 [IQR, 0.50–0.83]; *P* = .028), where high rates of inbreeding have been described in recent years [13, 33].

### High Diversity and Frequent Outcrossing in Ethiopia

**Figure 2.**
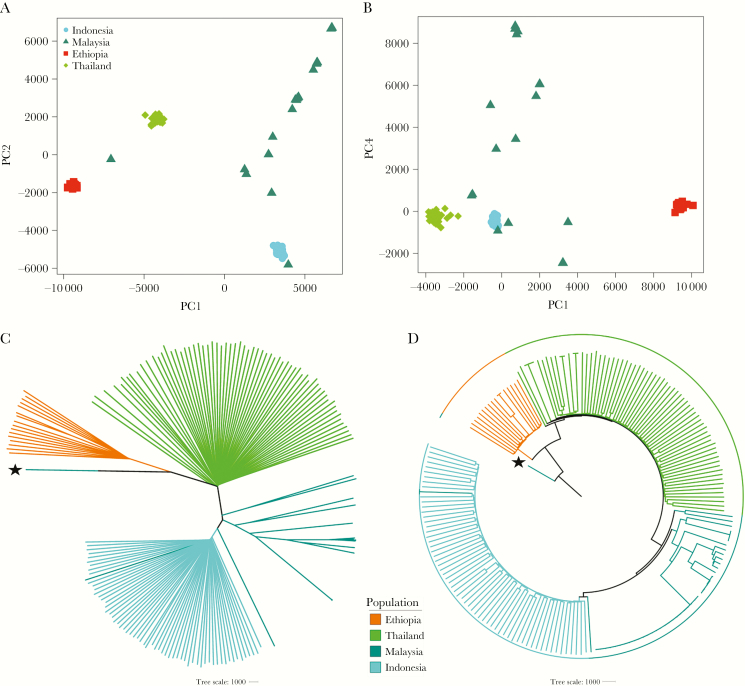
*Plasmodium vivax* population structure and relatedness in Ethiopia relative to the Asian populations. All plots were generated using genomic data derived from 191 high-quality, monoclonal (within-sample *F* statistic [*F*_WS_] > 0.95) samples. *A* and *B*, Principal coordinates analysis plots illustrating the genetic differentiation within and between populations, respectively. Principal components (PC) 1–4 reflect 16.8%, 10.8%, 9%, and 2.9% of the variance, respectively. *C* and *D*, Unrooted and rooted neighbor-joining trees, respectively. The rooted tree is presented to illustrate similarity between infections in a given population rather than evolutionary patterns. The PY0120-C isolate from Malaysia, labeled with a star, was used as the ancestral sample; this sample is a suspected imported case that has been shown to have close identity with infections from India and Bangladesh (data not presented).

The average nucleotide diversity (*pi*) in Ethiopia (6.47 × 10^-4^) was lower than Thailand (7.69 × 10^-4^) and Indonesia (6.78 × 10^-4^) but higher than Malaysia (4.55 × 10^-4^). In contrast to the extensive substructure observed in Malaysia with PCoA and NJ analysis, higher levels of outcrossing were apparent in Ethiopia, Thailand, and Indonesia (Figure 2A–D). Similar results were observed with the deconvoluted Ethiopian haplotypes (Supplementary Figure 3*A*–*D*).

### Varying Prevalence of Drug Resistance–Associated Mutations in Ethiopia

The prevalence of mutations associated with clinical or ex vivo antimalarial drug resistance was investigated in each population (Table 1). The multidrug resistance 1 (MDR1) Y976F variant, a minor modulator of CQ resistance [34], reached 32% (6/19) in Ethiopia, compared with 13% (14/104) in Thailand, 100% (111/111) in Indonesia, and 94% (51/54) in Malaysia. The prevalence of mutations in dihydrofolate reductase (DHFR) and dihydropteroate synthase (DHPS), which have been associated with antifolate resistance [34], was generally lower in Ethiopia than Asia. There were no DHFR triple or quadruple mutants (0% [0/18]) in Ethiopia, but 94% (17/18) of isolates had double mutations. Malaysia also exhibited a moderately low prevalence of triple and quadruple DHFR mutants (27% [14/51]), although these were prevalent in Thailand (99% [88/89]) and Indonesia (83% [77/93]). Ethiopia also exhibited a lower prevalence of DHPS A553G (0% [0/24]) and A383G (17% [4/24]) than the Asian populations (Table 1). None of the Ethiopian isolates had MDR1 CNVs. For reference purposes, the prevalence of nonsynonymous variants in orthologues of other genes implicated in drug resistance in *P. falciparum* is provided in Supplementary Data 3, including chloroquine resistance transporter (CRT), kelch-13, plasmepsin IV, multidrug resistance–associated proteins 1 and 2, and multidrug resistance protein 2.

**Table 1.** Prevalence of Orthologous Drug Resistance Markers at Each Site

Gene	Chr	Positions	Mutation	Drug	Frequency, % (no./No.)^a^			
					Ethiopia	Thailand	Indonesia	Malaysia
MDR1	10	479 908	F1076L	CQ	100 (24/24)	50 (54/104)	100 (111/111)	98 (53/54)
(PVP01_1010900)	10	480 207	Y976F	CQ, AQ + SP	32 (6/19)	13 (14/104)	100 (111/111)	94 (51/54)
	10	Copy number variant	≥2 copies	MQ	0 (0/24)	19 (20/104)	0 (0/84)	0 (0/49)
DHFR-TS	5	1 077 530; 1 077 532	F57L/I	Antifolate, AQ + SP	0 (0/24)	91 (88/97)	82 (77/94)	94 (48/51)
(PVP01_0526600)	5	1 077 533; 1 077 534; 1 077 535	S58R	Antifolate, AQ + SP	94 (17/18)	100 (104/104)	99 (104/105)	36 (19/53)
	5	1 077 543	T61M	Antifolate, AQ + SP	0 (0/24)	91 (90/99)	82 (77/94)	25 (13/51)
	5	1 077 711	S117N/T	Antifolate, AQ + SP	100 (24/24)	100 (102/102)	99 (93/94)	100 (53/53)
	…	…	Single mutant	Antifolate, AQ + SP	6 (1/18)	0 (0/89)	1 (1/93)	0 (0/51)
	…	…	Double mutant	Antifolate, AQ + SP	94 (17/18)	1 (1/89)	16 (15/93)	73 (37/51)
	…	…	Triple mutant	Antifolate, AQ + SP	0 (0/18)	0 (0/89)	0 (0/93)	2 (1/51)
	…	…	Quadruple mutant	Antifolate, AQ + SP	0 (0/18)	99 (88/89)	83 (77/93)	25 (13/51)
DHPS	14	1 270 401	A553G	Antifolate	0 (0/24)	98 (98/100)	16 (16/97)	91 (48/53)
(PVP01_1429500)	14	1 270 911	A383G	Antifolate	17 (4/24)	100 (104/104)	97 (106/109)	94 (50/53)

Mutation prevalence was calculated with homozygous calls only.

Abbreviations: AQ, amodiaquine; Chr, chromosome; CQ, chloroquine; MQ, mefloquine; SP, sulfadoxine-pyrimethamine.

^a^Genotype failures were <5% at all markers in all populations.

### Evidence of Recent Directional Selection on an AP2 Domain Transcription Factor in Ethiopia Using iHS Analysis

**Figure 3.**
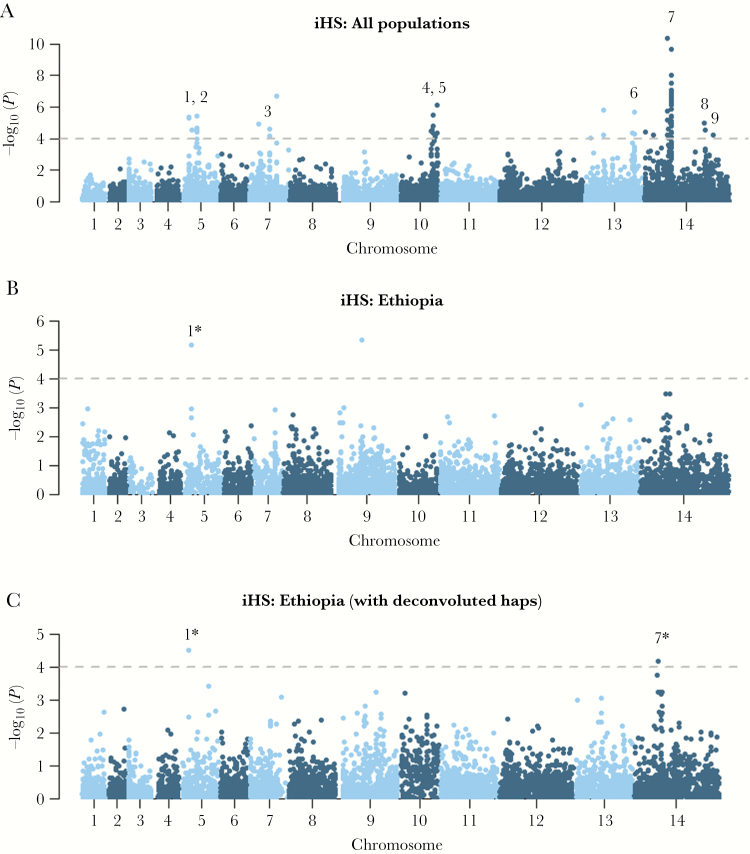
Genome-wide scans of extended haplotype homozygosity using the integrated haplotype score (iHS) illustrating regions under recent directional selection in Ethiopia and across populations. Manhattan plots of the iHS *P* value for the given populations: all monoclonal (within-sample *F* statistic [*F*_WS_] > 0.95) samples from Ethiopia, Thailand, and Indonesia (*A*), all monoclonal (*F*_WS_ > 0.95) samples from Ethiopia (*B*), and all monoclonal (*F*_WS_ > 0.95) samples from Ethiopia plus the 7 major haplotypes derived from polyclonal Ethiopian isolates that were deconvoluted (*C*) using DEploid identity by descent (IBD) software. Data are presented on 260 982 loci for which derived alleles could be confidently called. The dashed black lines demark the thresholds of –log_10_ (*P* value) > 4: signals supported by a minimum of 3 single-nucleotide polymorphisms (SNPs) above the threshold within 50 kb of one another and with an overall SNP density <10 kb per SNP are numbered. Details can be found in Supplementary Data 4. In brief, the putative genetic drivers include an SPRY domain protein (PVP01_0508000) (signal 1), 3 conserved proteins with unknown function (PVP01_0515000, PVP01_1029200, and PVP01_1455300) (signals 2, 4, and 9 respectively), gamma-glutamylcysteine synthetase (PVP01_0717300), an AP2 domain transcription factor (PVP01_1418100) or ferredoxin (PVP01_1419000) (signal 7), and an amino acid transporter (PVP01_1449600) (signal 8). The driver in the 92-kb region on chromosome 13 (signal 6) remains unclear. *Signals were supported by a minimum of 3 SNPs above the threshold in the population-wide data but only 1 SNP in Ethiopia.

Analysis of other gene regions demonstrating evidence of recent directional selection was undertaken using the iHS score. Population-wide analyses revealed 9 regions with strong signals of selection, 2 of which demonstrated weak signals in Ethiopia (Figure 3; Supplementary Data 4). The largest signal in Ethiopia was a 220-kb region on chromosome 14 comprising 50 genes and overlapping with a previously described signal postulated to reflect selection at an AP2 domain transcription factor (PVP01_1418100) [17]. The other Ethiopian signal reflected a 1-kb region on chromosome 5 comprising a SPRY domain protein (PVP01_0508000).

### *Rsb*-Based Signals of Differential Selection Between Ethiopia and Asia in Drug Resistance Candidates

**Figure 4.**
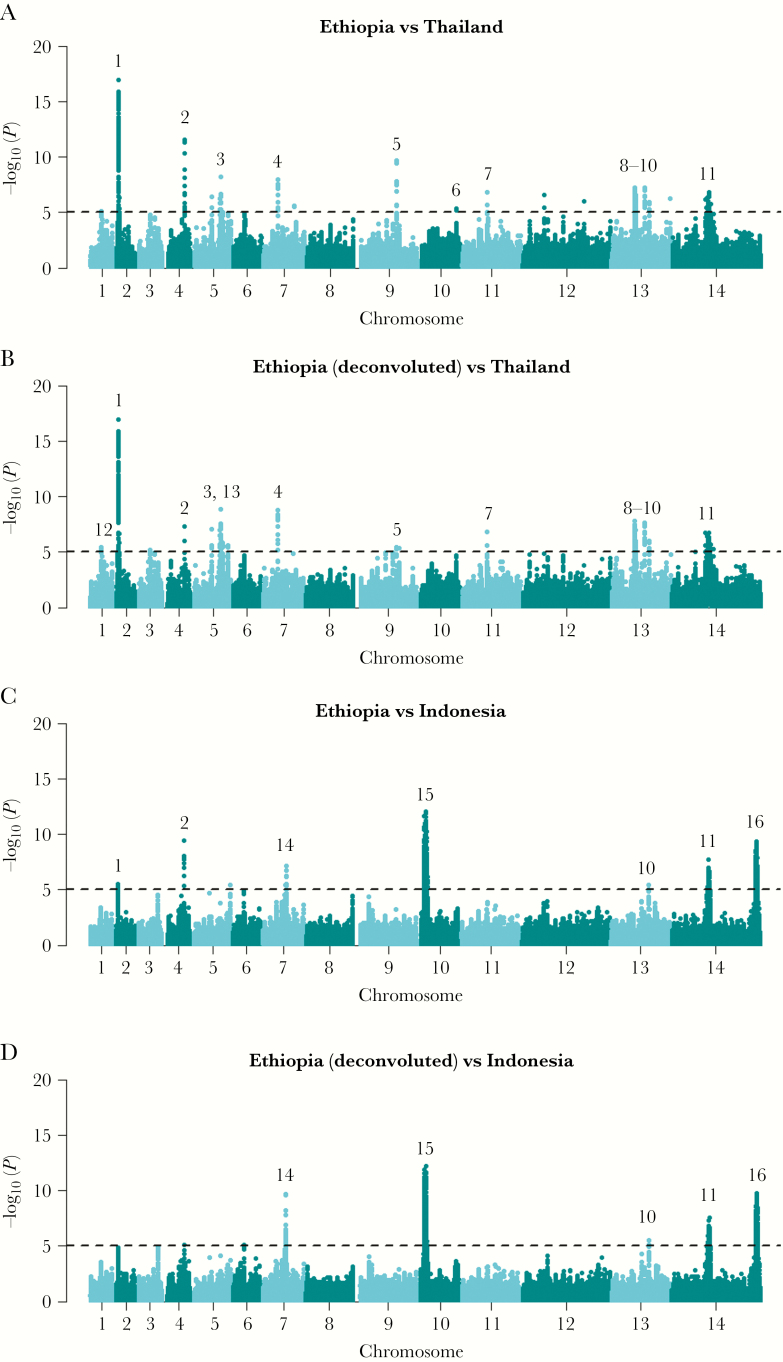
Genome-wide scans of *Rsb*-based cross-population extended haplotype homozygosity illustrating regions under divergent selection between Ethiopia and Thailand and Indonesia. *A–D*, Manhattan plots of the *Rsb P* value for the given populations. The dashed black lines demark the thresholds of –log_10_ (*P* value) > 5: signals supported by a minimum of 3 single-nucleotide polymorphisms (SNPs) above the threshold within 50 kb of one another and with an overall SNP density <10 kb per SNP are numbered. The multi-SNP signals are detailed in Supplementary Data 4. Several previously described signals in known or putative drug resistance candidates were identified including MRP1 (signal 1), DHFR (signal 3), and DHPS (signal 11). Signals were also observed in regions upstream of CRT (signal 12) and MDR1 (signal 15). The putative genetic drivers in other regions include MSP5 (signal 2), a tRNA (signal 4), AMA1 (signal 5), a merozoite surface protein 3 family cluster (signal 6), a voltage-dependent anion-selective channel (signal 8), liver-specific protein 1 (signal 10), and an AP2 domain transcription factor (signal 13). The putative genetic drivers in regions 14 and 16 are conserved *Plasmodium* proteins with unknown function, and those in regions 7 and 9 remain unclear.

The *Rsb* metric was used to identify regions with recent directional selection in one population relative to another. Sixteen regions demonstrated strong differential selection between Ethiopia and Thailand or Indonesia, including 5 drug resistance candidates (Figure 4; Supplementary Data 4). The DHFR and DHPS regions demonstrated greater extended haplotype homozygosity indicative of stronger selection in Thailand and Indonesia relative to Ethiopia. Strong signals were also observed on chromosome 2, reflecting MRP1 selection in Thailand and Indonesia, and a region upstream of MDR1 on chromosome 10 in Indonesia. The only drug resistance candidate with evidence of recent selection in Ethiopia was a region upstream of CRT, with extended haplotypes relative to Thailand. Signals of selection were observed in Ethiopia in other gene functions including pathogenesis (merozoite surface protein 5 [MSP5], apical membrane antigen 1 [AMA1]) and regulation of gene expression (AP2 domain transcription factor [PVP01_0529800], *tRNA* [PVP01_0711200]).

### *F*_ST_-Based Differentiation of Nonsynonymous Variants Between Ethiopia and Asia in Genes Involved in Regulation of Gene Expression

*F*_ST_ analysis was undertaken to identify recent as well as older signals of selection that might not be detected with haplotype-based methods. A total of 306 and 1162 SNPs exhibited *F*_ST_ > 0.8 in Ethiopia vs Thailand and Indonesia, respectively; among these variants, 126 and 461 conferred nonsynonymous changes (Supplementary Data 5). After Bonferroni correction, there was no significant representation of any GO terms; however, the most enriched class of genes with highly differentiated nonsynonymous variants were those involved in the regulation of gene expression (11/68 genes with GO ID 0010468). Among the known drug resistance–associated determinants, high differentiation was observed between Ethiopia and Thailand in the *DHPS* A383G mutation.

### Tajima *D* Analysis in Ethiopia

Tajima’s *D* analysis was conducted to identify regions under balancing as well as directional selection in Ethiopia (Supplementary Data 6). There was no significant enrichment of any GO term in either the 1st or 99th percentile of *D* scores in Ethiopia after Bonferroni correction. CRT and a proximal gene on chromosome 1 involved in ion transport (SCO1, PVP01_0109200) were among the 1st percentile in Ethiopia, suggesting evidence of positive selection. However, among 43 CRT and 15 SCO1 SNPs detected across the 3 populations, only 3 and 4 SNPs, respectively, appeared to be segregating in Ethiopia. Furthermore, neither CRT nor SCO1 were among the 1st percentile of genes in Thailand or Indonesia. A range of genes with various functions was observed among the 99th percentile in Ethiopia, reflecting genes putatively under balancing selection, including AMA1.

### High Prevalence of Duffy Binding Protein 1 Copy Number Amplifications in Ethiopia

**Figure 5.**
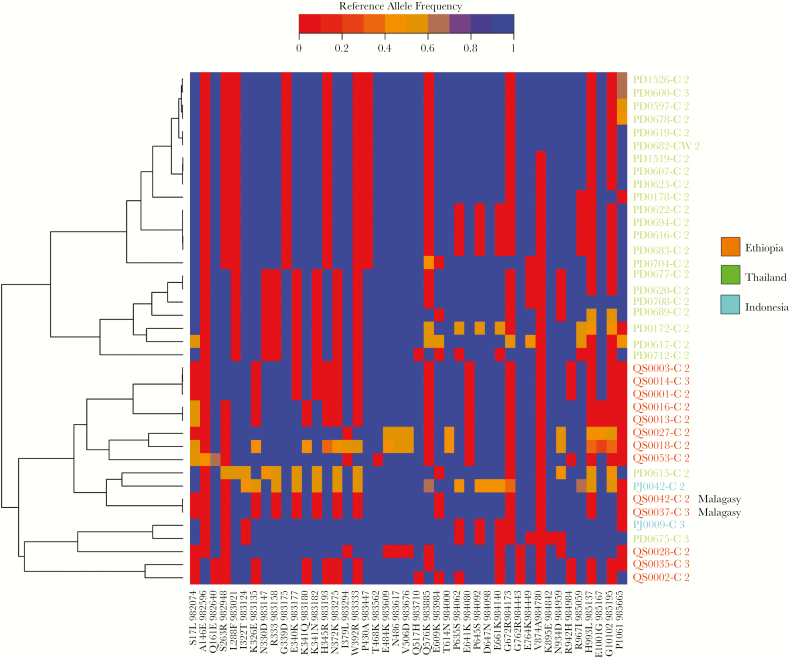
Heatplot illustrating multiple Duffy binding protein 1 (DBP1) haplotypes between samples and divergence between DBP1 copies within samples in monoclonal infections with copy number amplifications. The heat plot presents color-coded genotype calls at single-nucleotide polymorphisms in the DBP1 gene with minor allele frequency ≥1%. Genotypes are presented as reference allele frequencies ranging from 0 in red (homozygote alternative allele) to 1 in blue (homozygote reference allele). Samples are ordered on the y-axis according to their genetic relatedness as per the left-hand phylogram. Sample labels are color-coded according to country. Only monoclonal (within-sample *F* statistic > 0.95) infections with ≥2 DBP1 copies were included in the analyses. Therefore, heterozygous positions (in orange) reflect differences between the DBP1 copies within a given infection. Two Malagasy-type DBP1 amplifications are labeled; all other amplifications were Cambodian type.

Copy number amplification was observed in 3 gene regions in Ethiopia; a 28S ribosomal RNA gene (PVP01_0504500) with amplification in 100% infections, an exported *Plasmodium* protein (PVP01_1470400) in 50% infections, and Duffy binding protein 1 (DBP1, PVP01_0623800) in 79% infections (Table 2; Supplementary Data 7). Deletions were also observed in 2 regions encompassing exported *Plasmodium* proteins: PVP01_0523500 present in 63% (15/24) of Ethiopian isolates and PVP01_0524900 + PVP01_0525000 in 4% (1/24). While the deletions were only observed in Ethiopia, the amplifications were also observed in Asia. The genetic architecture of the DBP1 amplification, which is putatively involved in *P. vivax* invasion of Duffy-negative RBCs [35], was explored further. DBP1 copy number ranged from 2 to 5 in Ethiopia, and 2 to 3 in the Asian populations. Artemis-based inspection of the amplification revealed a single 5′ and two 3′ breakpoints reflecting the previously described Malagasy and Cambodian type breakpoints [36, 37] in Ethiopia (Supplementary Figure 4). The most common breakpoint was the Cambodian type (63% [16/19]), also common in Indonesia (100% [5/5]) and Thailand (96% [30/31]). Inspection of the monoclonal infections with amplifications revealed a large range of DBP1 haplotypes in Ethiopia (minimum 8 haplotypes based on differences at nonheterozygous positions) (Figure 5). Furthermore, 38% (5/13) of isolates exhibited haplotype differences (heterozygote positions) between the copies within a given infection. The Thai parasites formed a largely separate cluster from Ethiopia, but with similar patterns of DBP1 diversity within and between infections (minimum 12 haplotypes, 35% [7/20] infections with heterozygotes). The sequence flanking DBP1 also displayed moderate diversity in Ethiopia and Asia, with no evidence of selective sweep dynamics (Supplementary Figure 5).

**Table 2.** Summary of Copy Number Variants

Gene	Description	Gene Coordinates	Variant Type	Frequency, % (no./No.)			
				Ethiopia	Thailand	Indonesia	Malaysia
PVP01_0504500	28S ribosomal RNA	5: 194 522-1 949 297	Amplification	100 (24/24)	92 (96/104)	95 (80/84)	89 (41/46)
PVP01_0523500	*Plasmodium* exported protein	5: 951 202–952 041	Deletion	63 (15/24)	0 (0/104)	0 (0/84)	0 (0/46)
PVP01_0524900, PVP01_0525000	*Plasmodium* exported proteins	5: 1 010 759-1 011 581 5: 1 013 733-1 014 550	Deletion	4 (1/24)	0 (0/104)	0 (0/84)	0 (0/46)
PVP01_0623800	Duffy binding protein 1	6: 982 015–985 813	Amplification	79 (19/24)	30 (31/104)	6 (5/84)	4 (2/46)
PVP01_1470400	*Plasmodium* exported protein	14: 3 009 446-3 010 524	Amplification	33 (8/24)	49 (51/104)	46 (39/84)	89 (41/46)

Twenty-two samples with an excess of copy number variants (≥18) were excluded from analysis.

## DISCUSSION

This study presents the first population genomic investigation of *P. vivax* in Ethiopia, where the largest reservoir of this species in Africa persists. Our results reveal patterns of polyclonality, within-infection relatedness, population diversity, and structure comparable to Thailand and Indonesia, where transmission was stable at the time of sampling. We see evidence of genetic adaptations in various parasite mechanisms, which may facilitate the ongoing persistence of the population. The different forms of selection and adaptive responses that appear to be operating in the population are discussed further.

The striking prevalence of DBP1 amplification implies an important adaptive role in the host blood stage of infection in Ethiopia. The amplification has been found at low frequency in Malaysia (4%) and Indonesia (6%), and at higher frequencies in Cambodia (29%), Thailand (31%), and Madagascar (53%) [5, 13, 36, 37]. The prevalence in Ethiopia reached almost 80%, higher than in any population examined to date [5, 13, 36, 37]. The high haplotype diversity in the flanking regions of the DBP1 amplifications implies that it has most likely arisen independently on multiple occasions without being purged from the population. In a previous study in Thailand, a highly prevalent MDR1 amplification that conferred resistance to mefloquine was rapidly purged from the population after the drug was discontinued [24],. The fitness consequence of the DBP1 amplification is less clear. A genetic epidemiology study of DBP1 amplification in Cambodia speculated that it may have an immune-related role by enhancing antigenic diversity on the RBC membrane [36]. Our assessments in Ethiopia and Thailand also revealed moderately high DBP1 haplotype diversity, with haplotypic differences between copies enriching the diversity in >35% of monoclonal infections. However, the results of a recent functional study imply that the amplification may enhance parasite binding to Duffy-negative reticulocytes using a Duffy-independent invasion pathway [35]. Whether functioning in immune evasion or Duffy-negative reticulocyte invasion, the high prevalence of the DBP1 amplification in Ethiopia warrants close monitoring of this variant across the continent.

Antimalarial drug resistance presents another challenge to the containment of vivax in the Horn of Africa. Chloroquine (CQ) remains the first-line treatment for *P. vivax* blood-stage infection in Ethiopia, but recent surveys have demonstrated declining CQ efficacy against *P. vivax*, with recurrence ranging from 4% to 22% by day 28 [38–41]. Although the prevalence of the MDR1 Y976F mutation in Ethiopia (32%) was not as high as that documented in Papua Indonesia or Malaysia, where high-grade CQ resistance (CQR) has been reported [42, 43], ongoing surveillance is needed. Furthermore, since MDR1 Y976F mutation is not a prerequisite for high-grade CQR [34], additional markers are needed to facilitate these efforts. Several studies have explored the role of *P. vivax* CRT, the orthologue of PF3D7_0709000, the primary determinant of CQR in *P. falciparum* [44]; however, results are inconsistent [45–49]. Interestingly, we found evidence of extended haplotype homozygosity in Ethiopia relative to Thailand in a region upstream of CRT, potentially reflecting selection on a regulator of CRT expression in Ethiopia. While studies in Brazil have reported association between CRT expression and CQR in *P. vivax* using clinical phenotypes [46, 47], the lack of association using ex vivo phenotypes in isolates from Papua Indonesia [45], the epicenter of CQR for this species [50], is not consistent with a pivotal role. However, it is possible that different variants are operating in different populations. Further studies are warranted.

The absence of MDR1 amplification in Ethiopia suggests that mefloquine may be an appropriate treatment alternative for vivax, should CQR continue to rise. However, the high prevalence of DHFR double mutants (94%) raises concerns for pyrimethamine in regimens such as intermittent preventive treatment for pregnant women or infants. Interestingly, in contrast to the Asian populations, there were no triple or quadruple DHFR mutants and <20% prevalence of DHPS resistance-conferring mutants in Ethiopia, reflecting lower selective pressure. Accordingly, the haplotype-based signals of differential selection at the DHFR and DHPS loci reflected directional selection patterns in Thailand and Indonesia, not Ethiopia. It has been postulated that drug resistance variants may emerge more readily in populations with high rates of inbreeding. We did not find evidence of any significant difference in within-infection relatedness between Ethiopia and the Thai or Indonesian populations, implying comparable levels of inbreeding between these populations. Alternatively, differences in drug history or drug usage, which is generally postulated to be higher in Asia than Africa, might have contributed to lower selective pressure in Ethiopia.

Some of the strongest signals of selection in Ethiopia were observed in MSP5 and AMA1. Tajima *D* analysis demonstrated evidence of balancing selection in AMA1, consistent with high antigenic variation facilitating evasion of host immune pressure on the RBC stage of the parasite. Both AMA1 and MSP5 also exhibited evidence of differential selection between populations, reflecting complex haplotype structures of different strains circulating in different populations.

We also found evidence of adaptive variation in genes involved in regulation of gene expression, including several AP2 domain transcription factors. One of the strongest signals reflected an AP2 transcription factor on chromosome 14 (PVP01_1418100) under directional selection in Ethiopia and Asia. A previous study in Cambodia also reported strong selection on PVP01_1418100 and other transcription factors, highlighting the importance of these regulatory determinants in modulating mechanisms critical to the transmission and maintenance of vivax populations such as gametocytogenesis and hypnozoite dormancy and activation [17].

Although pressure from antimalarial drugs appears to be weaker in Ethiopia than Southeast Asia, the evidence of selection in genes involved in a variety of processes at various parasite life-cycle stages calls for multifaceted intervention approaches to contain *P. vivax* in the region. Ongoing genetic and genomic surveillance will aid in the detection of new forms of adaptation as they arise in the parasite population.

## Supplementary Data

Supplementary materials are available at *The Journal of Infectious Diseases* online. Consisting of data provided by the authors to benefit the reader, the posted materials are not copyedited and are the sole responsibility of the authors, so questions or comments should be addressed to the corresponding author.

jiz016_suppl_Supplementary_Figure_1

jiz016_suppl_Supplementary_Figure_2

jiz016_suppl_Supplementary_Figure_3

jiz016_suppl_Supplementary_Figure_4

jiz016_suppl_Supplementary_Figure_5

jiz016_suppl_Supplementary_Data-1

jiz016_suppl_Supplementary_Data-2

jiz016_suppl_Supplementary_Data-3

jiz016_suppl_Supplementary_Data-4

jiz016_suppl_Supplementary_Data-5

jiz016_suppl_Supplementary_Data-6

jiz016_suppl_Supplementary_Data-7

jiz016_suppl_Supplementary_Material

## Notes

***Acknowledgments.*** We thank the patients who contributed their samples to the study; the health workers and field teams who assisted with the sample collections; the staff of the Wellcome Sanger Institute Sample Logistics, Sequencing, and Informatics facilities for their contributions; and the Director General of Health, Malaysia, for permission to publish this study.

***Financial support.*** The sample and metadata collection in Ethiopian and Indonesia were supported by a Wellcome Trust Senior Research Fellowship in Clinical Science (grant number 200909 to R. N. P.); the National Health and Medical Research Council of Australia (Improving Health Outcomes in the Tropical North: A Multidisciplinary Collaboration “Hot North” Career Development Fellowship , grant number 1131932 to S. A.); and the Bill & Melinda Gates Foundation (award number OPP1164105). The patient sampling and metadata collection for Malaysia were funded by the Asia-Pacific Malaria Elimination Network (award number 108-07), the Malaysian Ministry of Health (award number BP00500420), and the National Health and Medical Research Council of Australia (award numbers 1037304 and 1045156; fellowships to N. M. A. [award numbers 1042072 and 1135820], B. E. B. [award number 1088738] and M. J. G. [award number 1074795]). M. J. G. was also supported by a National Health and Medical Research Council of Australia “Hot North” Early Career Fellowship (award number 1131932). The whole genome sequencing component of the study was supported by grants from the Medical Research Council and UK Department for International Development (award number M006212) and the Wellcome Trust (award numbers 206194 and 204911) to D. P. K. In addition, S. J. Z. and G. M. were funded by the Wellcome Trust (grant number 100956/Z/13/Z to G. M.). This work was supported by the Australian Centre for Research Excellence on Malaria Elimination (ACREME), funded by the National Health and Medical Research Council of Australia (APP 1134989).

***Potential conflicts of interest.*** All authors: No reported conflicts of interest. All authors have submitted the ICMJE Form for Disclosure of Potential Conflicts of Interest. Conflicts that the editors consider relevant to the content of the manuscript have been disclosed.
