# Supplementary figures and images for: Genomic Analysis of Plasmodium vivax in Southern Ethiopia Reveals Selective Pressures in Multiple Parasite Mechanisms
Source: J Infect Dis. 2019 Jan 21;220(11):1738–49. doi: 10.1093/infdis/jiz016 (PMC6804337; doi:10.1093/infdis/jiz016)

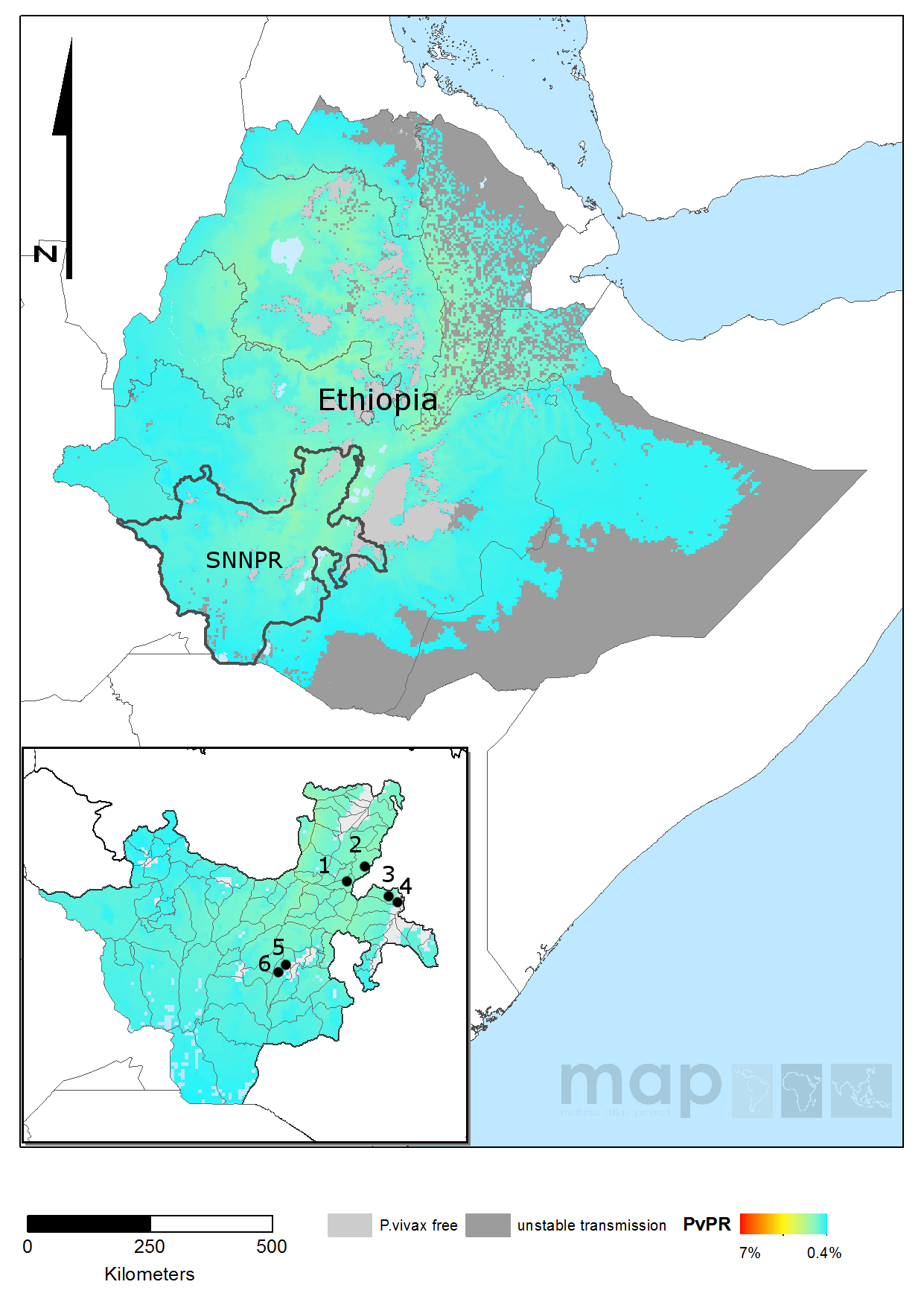

Supplement: jiz016_suppl_Supplementary_Figure_1 [file jiz016_suppl_supplementary_figure_1.png]

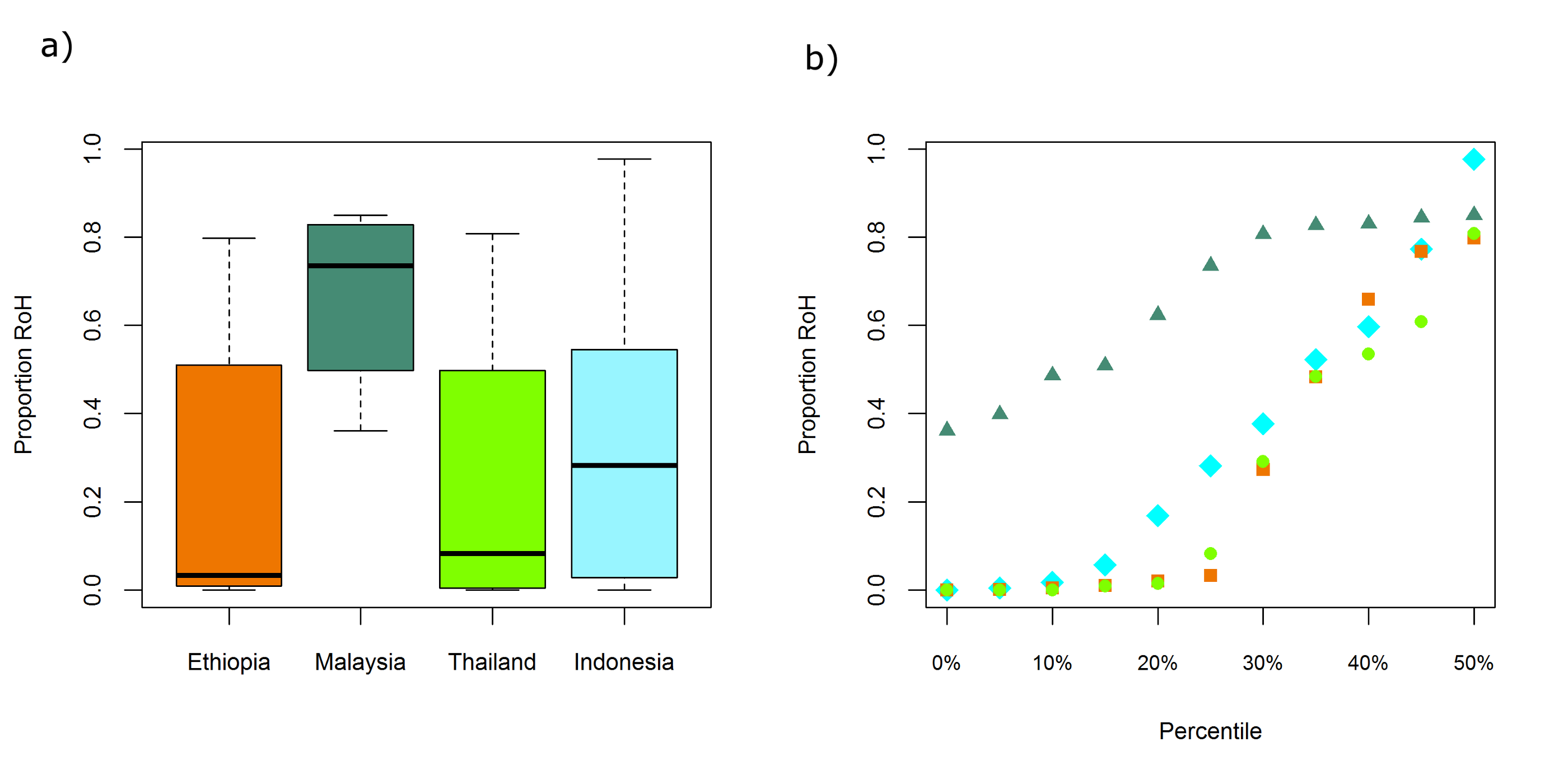

Supplement: jiz016_suppl_Supplementary_Figure_2 [file jiz016_suppl_supplementary_figure_2.png]

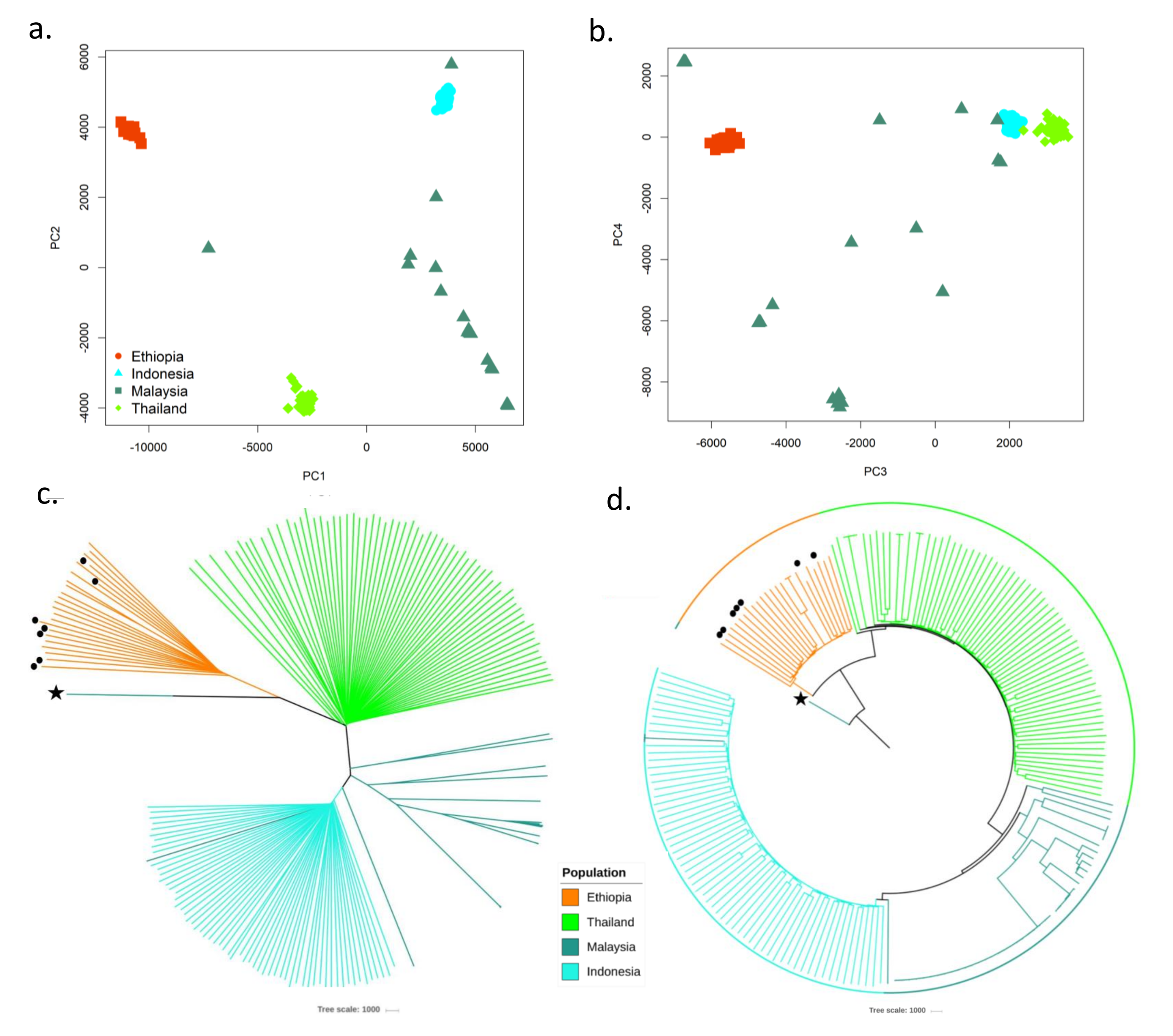

Supplement: jiz016_suppl_Supplementary_Figure_3 [file jiz016_suppl_supplementary_figure_3.png]

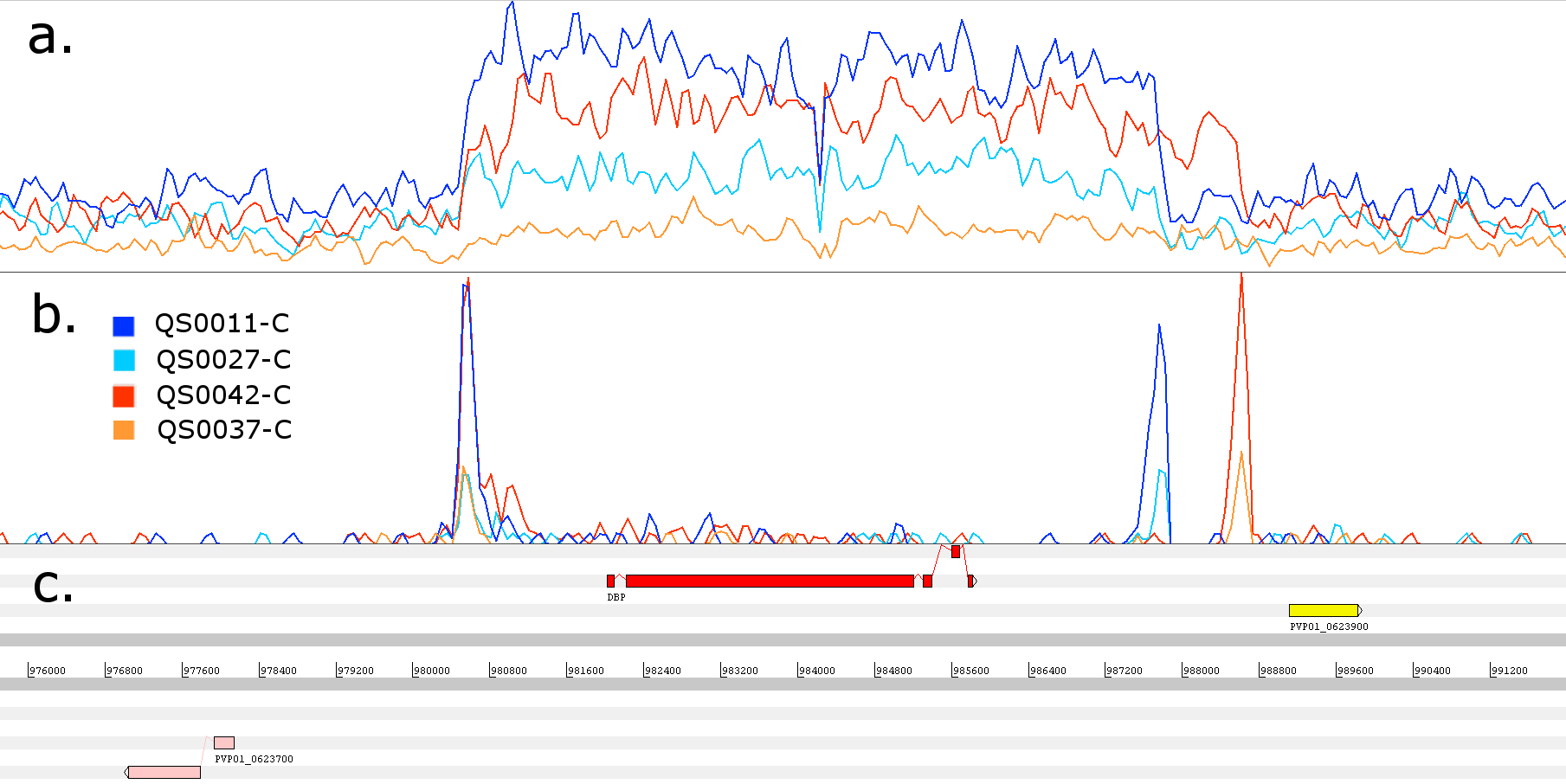

Supplement: jiz016_suppl_Supplementary_Figure_4 [file jiz016_suppl_supplementary_figure_4.png]

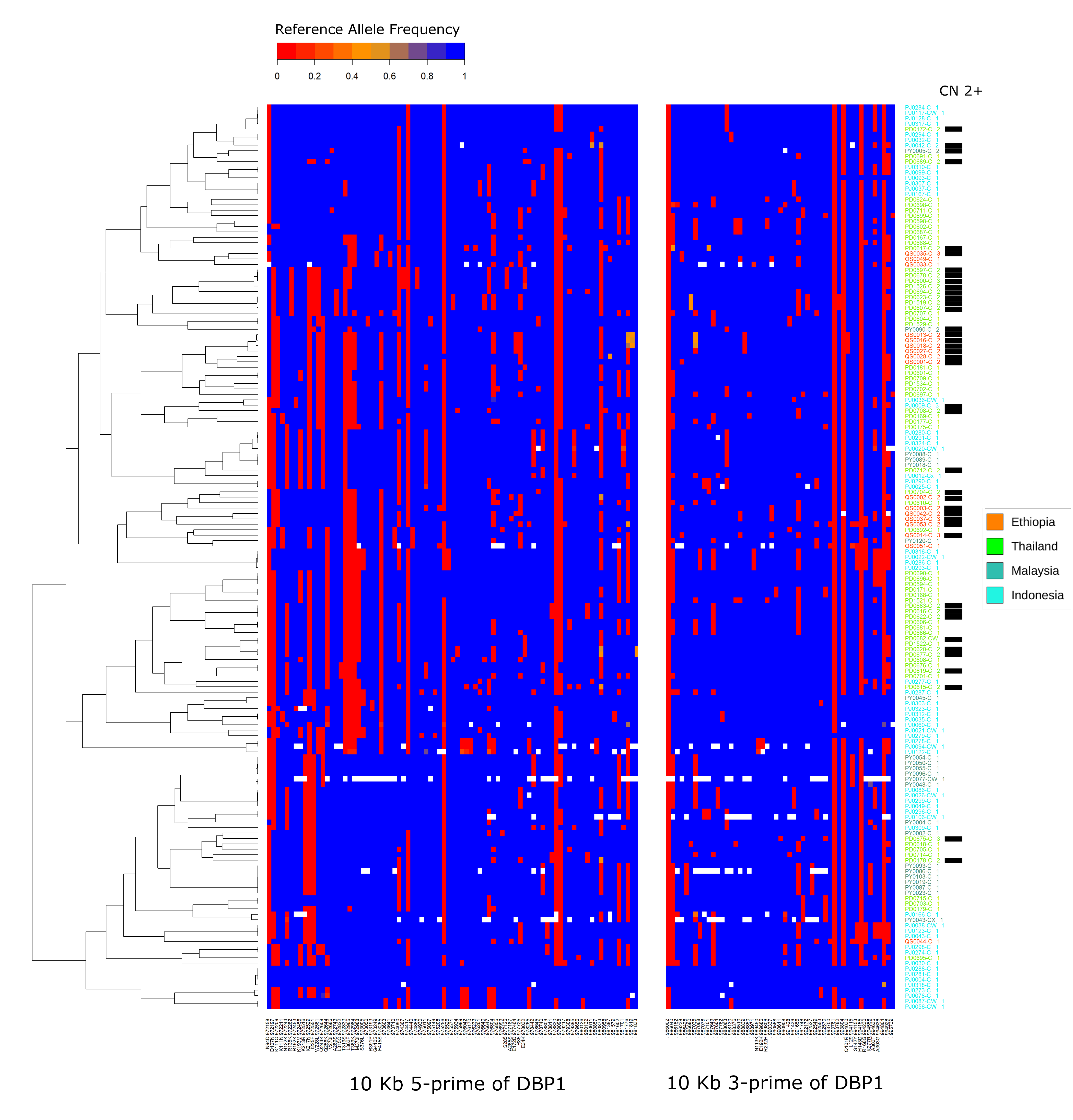

Supplement: jiz016_suppl_Supplementary_Figure_5 [file jiz016_suppl_supplementary_figure_5.png]
