# Supplementary material for: Genomic Analysis of Plasmodium vivax in Southern Ethiopia Reveals Selective Pressures in Multiple Parasite Mechanisms
Source: J Infect Dis. 2019 Jan 21;220(11):1738–49. doi: 10.1093/infdis/jiz016 (PMC6804337; doi:10.1093/infdis/jiz016)
Supplement: jiz016_suppl_Supplementary_Material [file jiz016_suppl_supplementary_material.docx]

**Supplementary Material**

**Supplementary Data 1. Summary of Ethiopian samples with high-quality genomic data**

**Supplementary Data 2. Measures of infection complexity and IBD**

*F*_WS_ and proportion of Runs of Homozygosity (RoH) for each infection from each of the four populations, and pairwise IBD derived by Deploid-IBD analysis for each of the seven polyclonal Ethiopian infections (isolates with *F*_WS_<0.95).

**Supplementary Data 3. Prevalence of non-synonymous variants in orthologues of *P. falciparum* drug resistance candidates**

Amino acid = amino acid change. Ref = reference. Alt = alternative. Alt 1 = alternative 1 (for tri-allelic positions). *Single base deletions. ** Positions with >20% genotyping failures or heterozygote calls. Positions with reference allele frequency below 75% are presented in yellow to highlight positions with moderate to high variability between populations.

**Supplementary Data 4. Summary of multi-SNP regions with evidence of *iHS* or *Rsb*-based signals of selection**

^a^ ET = Ethiopia, ET-D = Ethiopia with addition of deconvoluted haplotypes, TH = Thailand, ID = Indonesia. ^b^ Genomic regions supported by 3 or more SNPs with -log_10_(P-value)> 4 within 50 Kb of one another and with an overall SNP density less than 10 Kb per SNP. ^c^ Population with evidence of relatively extended haplotype homozygosity (labelling as per ^a^).

**Supplementary Data 5. Summary of positions with *F*_ST_≥0.8**

*F*_ST_ scores greater than or equal to 0.8 are highlighted in green. ET = Ethiopia, ET-D = Ethiopia with addition of deconvoluted haplotypes, TH = Thailand, ID = Indonesia, and MY = Malaysia. Amino acid = amino acid change; “.” refers to intergenic sites. Ref = reference allele, Alt = Alternate allele, RAF = reference allele frequency.

**Supplementary Data 6. Genes amongst the top 1 and 99 percentile of Tajima’s *D* scores.** Tajima’s *D* results and number of SNPs are presented for the genes in the top 1 and 99 percentiles in the respective populations; ET (monoclonal Ethiopian samples (*F*_WS_>0.95)), ET-D (monoclonal Ethiopian samples plus the major deconvoluted haplotypes from polyclonal infections, TH (monoclonal Thai samples), and ID (monoclonal Indonesian samples). Owing to extensive population structure, results are not presented for Malaysia. Only genes with a minimum of 10 SNPs across the pooled Ethiopian and Asian samples were assessed, amounting to 3,861 genes in total.

**Supplementary Data 7. Summary of copy number variants**

**Supplementary Figure 1. Study site in Ethiopia.** This figure (also presented in [10]) provides a *P. vivax* prevalence map for Ethiopia, with a zoomed in view of the South Nations Nationalities and People’s Region (SNNPR), generated by the Malaria Atlas Project. The colour scales reflect the model-based geostatistical point estimates of the annual mean *P. vivax* parasite rate in the 1-99 year age range (*Pv*PR_1-99_) within the stable spatial limits of transmission in 2010. All Malaria Atlas Project maps are available to users under the Creative Commons Attribution License CCAL 3.0. [http://www.map.ox.ac.uk/about-map/open-access/]. The numbered dots in the SNNPR panel illustrate the locations of the health centers from which samples were recruited; Shone Health Center, Badawacho (1), Guba Health Center, Halaba (2), Adare Hospital, Hawassa (3), Millenium Health Center, Hawassa (4), Arbaminch Hospital, Arbaminch (5), and Shele Health Center, Arbaminch (6). The maximum distance between sites is 180 Km (between Guba Health Center (2) and Shele Health Center (6)).

**Supplementary Figure 2. Proportions of Runs of Homozygosity in polyclonal infections from Ethiopia relative to the Southeast Asian populations.** The boxplots (panel a) and scatter plots (panel b) illustrate the distribution of RoH scores in Ethiopia relative to Thailand, Indonesia and Malaysia. Data is presented on the polyclonal infections only (*F*_WS_<0.95). The RoH in the polyclonal Malaysian samples is higher than the other populations, indicating higher rates of co-transmission of half or full-siblings versus superinfections in this low transmission setting.

**Supplementary Figure 3. *P. vivax* population structure and relatedness with inclusion of the deconvoluted Ethiopian samples.** All plots were generated using genomic data derived from 191 high-quality, monoclonal (*F*_WS_>0.95) samples and 7 major haplotypes derived from polyclonal Ethiopian isolates that were deconvoluted using DEploid-IBD software. Panels a) and b) present PCoA plots illustrating the genetic differentiation within and between populations. Principal Components 1-4 reflect 20.6%, 10.2%, 9.3% and 2.5% of the variance respectively. Panels c) and d) illustrate unrooted and rooted neighbour-joining trees respectively. The PY0120-C isolate from Malaysia, labelled with a star, was used as the ancestral sample; this samples is a suspected imported case that has been shown to have close identity with infections from India and Bangladesh (data not presented). The deconvoluted Ethiopian haplotypes are indicated with black dots.

**Supplementary Figure 4. Illustration of the DBP1 breakpoint regions in the Ethiopian isolates.**

Panel a) illustrates the depth (height) of correctly mapping reads in examples of four Ethiopian isolates with 2+ DBP1 copies. The isolates represent the range of DBP1 amplicon sizes determined by the hidden markov model-based CN detection algorithm; QS0027-C = 6,900 bp, QS0011-C = 7,200 bp, QS0037-C = 8,100 bp and QS0042-C = 9,600 bp. Panel b) presents the depth (height) of faceaway mapping reads in the same samples illustrating the breakpoint regions. Panel c) illustrates the genomic features in the region. As illustrated by the peak read depth of faceaway reads, all four of the isolates had the same 5’ breakpoint (Cambodian and Malagasy type 5’ breakpoint). QS0011-C and QS0027-C (6,900-7,200 bp) have the Cambodian type 3’ breakpoint, while QS0037-C and OS0042-C (8,100-9,600 bp) have the Malagasy type 3’ breakpoint.

**Supplementary Figure 5. Heatplot illustrating the relatedness in the DBP1 flanking regions between isolates with one versus multiple gene copies.**

The heatplot presents colour-coded genotype calls at SNPs in the regions 10 Kb 5-prime and 3-prime of the DB1 gene with MAF>=1%. Genotypes are presented as reference allele frequencies ranging from 0 in red (homozygote alternative allele) to 1 in blue (homozygote reference allele). Samples are ordered on the y-axis according to their genetic relatedness as per the left-hand phylogram. Sample labels are colour-coded according to country. The black bars adjacent to the sample labels highlight samples with 2+ DBP1 copies. Only monoclonal (*F*_WS_>0.95) infections were included in the analyses. Only a single representative of the large cluster of identical Malaysian isolates was included in the analysis.
